# Risk Factors for Subjective Memory Concern Endorsement in Ethnoracially Diverse Middle and Late Life Adults

**DOI:** 10.1002/alz70860_106011

**Published:** 2025-12-23

**Authors:** Christopher A. Krause, Alexa S. Gonzalez, Luis D Medina

**Affiliations:** ^1^ University of Houston, Houston, TX, USA; ^2^ Baylor College of Medicine, Houston, TX, USA

## Abstract

**Background:**

Subjective memory concerns (SMC) may contribute to early identification of Alzheimer's disease and related (ADRDs) at prodromal stages. The Lancet report recently highlighted evidence for early, middle, and late life risk factors for ADRDs, including the addition of low‐density lipoprotein cholesterol (LDL‐c). However, the relationship between these risk factors and early markers of cognitive decline in ethnoracially diverse populations is still underexplored. We sought to elucidate the relationship between previously identified cognitive aging risk factors and SMCs in a diverse sample of adults.

**Method:**

Data from Hispanic/Latin American (*n* = 1023; 64% Spanish‐preferring) and non‐Hispanic Black (*n* = 570) participants in the Health & Aging Brain Study – Health Disparities (HABS‐HD) were analyzed (Age=62.51±7.78; Education = 12.9±4.64). Nested regression models were used to examine the impact of risk factors on SMC endorsement. Analyses were stratified by age (i.e., individuals ≤65 and >65), and by ethnoracial status/language.

**Result:**

In fully adjusted models stratified by age, history of traumatic brain injury (*b* = 2.067, *p* = 0.027, 95% CI: [0.235, 3.890], partial R^2^=0.005, *p* <0.001) and diabetes (*b* = 0.467, *p* = 0.034, 95% CI: [0.279, 0.340], partial R^2^=0.004, *p* <0.001) explained a small, yet significant proportion of variance in SMC endorsement for adults ≤65. Smoking status (*b* = ‐1.552, *p* = 0.002, 95% CI: [‐2.513, ‐0.592]) explained a small, yet significant proportion of variance (partial R^2^=0.018, *p* <0.001) unique to adults >65. In fully adjusted models stratified by ethnoracial status/language, LDL‐c (*b* = ‐0.012, *p* = 0.011, 95% CI: [‐0.012, ‐0.003]; partial R^2^=0.017, *p* <0.001) was uniquely predictive of SMCs in English‐speaking H/Ls, while traumatic brain injury (*b* = 2.953, *p* <0.001, 95% CI: [1.309, 4.597]; partial R^2^=0.022, *p* <0.001) and diabetes (*b* = 0.560, *p* = 0.030, 95% CI: [0.053, 1.067]; partial R^2^=0.008, *p* <0.001) were unique to non‐Hispanic Black participants. Depressive symptoms were a consistent and significant predictor of SMC endorsement across all models (partial R^2^ range: 0.281 to 0.304, all *p*s <0.001).

**Conclusion:**

Current findings demonstrate risk factors for SMCs in an ethnoracially diverse sample. The impact of these risk factors was modified by age and ethnoracial status/language. Additional forces (e.g., nonmedical determinants of health) may better explain and contextualize these findings. Results from this study may help inform future precision medicine efforts.

